# Prediction of depressive symptoms severity based on sleep quality, anxiety, and gray matter volume: a generalizable machine learning approach across three datasets

**DOI:** 10.1016/j.ebiom.2024.105313

**Published:** 2024-09-09

**Authors:** Mahnaz Olfati, Fateme Samea, Shahrooz Faghihroohi, Somayeh Maleki Balajoo, Vincent Küppers, Sarah Genon, Kaustubh Patil, Simon B. Eickhoff, Masoud Tahmasian

**Affiliations:** a Institute of Medical Science and Technology, Shahid Beheshti University, Tehran, Iran; b Institute of Systems Neuroscience, Medical Faculty, Heinrich Heine University Düsseldorf, Düsseldorf, Germany; c Institute of Neuroscience and Medicine, Brain & Behaviour (INM-7), Research Centre Jülich, Jülich, Germany; d Department of Nuclear Medicine, University Hospital and Medical Faculty, University of Cologne, Cologne, Germany

**Keywords:** Depressive symptoms severity, Sleep quality, Anxiety, Brain, Machine learning

## Abstract

**Background:**

Depressive symptoms are rising in the general population, but their associated factors are unclear. Although the link between sleep disturbances and depressive symptoms severity (DSS) is reported, the predictive role of sleep on DSS and the impact of anxiety and the brain on their relationship remained obscure.

**Methods:**

Using three population-based datasets (N = 1813), we trained the machine learning models in the primary dataset (N = 1101) to assess the predictive role of sleep quality, anxiety problems, and brain structural (and functional) measurements on DSS, then we tested our models’ performance in two independent datasets (N = 378, N = 334) to test the generalizability of our findings. Furthermore, we applied our model to a smaller longitudinal subsample (N = 66). In addition, we performed a mediation analysis to identify the role of anxiety and brain measurements on the sleep quality and DSS association.

**Findings:**

Sleep quality could predict individual DSS (r = 0.43, R^2^ = 0.18, rMSE = 2.73), and adding anxiety, contrary to brain measurements, strengthened its prediction performance (r = 0.67, R^2^ = 0.45, rMSE = 2.25). Importantly, out-of-cohort validations in other cross-sectional datasets and a longitudinal subsample provided robust similar results. Furthermore, anxiety scores, contrary to brain measurements, mediated the association between sleep quality and DSS.

**Interpretation:**

Poor sleep quality could predict DSS at the individual subject level across three datasets. Anxiety scores not only increased the predictive model's performance but also mediated the link between sleep quality and DSS.

**Funding:**

The study is supported by Helmholtz Imaging Platform grant (NimRLS, ZTI-PF-4-010), the 10.13039/501100001659Deutsche Forschungsgemeinschaft (DFG, GE 2835/2–1, GE 2835/4-1), the 10.13039/501100001659Deutsche Forschungsgemeinschaft (DFG, German Research Foundation)—Project-ID 431549029—SFB 1451, the programme “Profilbildung 2020” (grant no. PROFILNRW-2020-107-A), an initiative of the Ministry of Culture and Science of the State of Northrhine Westphalia.

**Research in context**

**Evidence before this study**

Depressive symptoms and clinical depression are prevalent in modern societies. Although the associated factors of clinical depression are well-documented, the predictive power of those factors in depressive symptoms in the general population is not well-identified. Several studies suggested that sleep disturbance and anxiety are linked with depressive problems in the general population and patients with major depressive disorder. A few meta-analyses and longitudinal studies also indicated that sleep disturbance plays a key role in developing depressive problems and clinical depression. However, those studies mainly used conventional group comparison statistical approaches, ignoring the inter-individual variability across participants. Moreover, their data were limited to a single database, limiting the generalizability of their findings in other samples. Thus, large-scale multi-sample studies using machine learning predictive approaches are needed to identify the complex pattern between sleep quality, anxiety problems, and depressive symptoms at the individual subject level. We also assessed the neurobiological underpinning of their interplay.

**Added value of this study**

In this study, we used machine learning, which enables individual-level predictions and can validate the generalizability of models on independent data. Thus, this analytical framework explicitly evaluates the generalization of trained models to new/unseen data. This study used three independent datasets, including three cross-sectional samples and a longitudinal subsample. We also performed careful complementary analyses to examine the robustness of our findings considering the impact of a lifetime history of depression, the effects of sleep-related questions of the depressive assessment, exploring the most important parameters of sleep quality in the prediction of depressive symptoms severity, and testing the reverse direction i.e., predicting sleep quality based on depressive symptoms. We found that poor sleep quality could robustly predict depressive symptoms across three datasets, but the reverse direction (prediction of sleep quality based on depressive symptoms) was less robust. Anxiety scores improved the performance of the predictive model and mediated the link between sleep and depressive symptoms, whereas our brain structural (and functional) properties were not able to predict depressive symptoms in this study. Our longitudinal assessment suggests that future depressive symptoms severity may be predictable based on baseline sleep and anxiety data.

**Implications of all the available evidence**

As depressive symptoms have a substantial impact on public health, identifying their contributing factors, such as poor sleep and anxiety, is critical to decreasing the burden of depressive symptoms and/or designing better therapeutical approaches at the individual subject level, which is essential toward precision medicine.

## Introduction

In modern societies, about 25% of the general population presents depressive symptoms such as sadness, irritability, anhedonia, low motivation, distracted concentration, worthlessness, abnormal appetite, and sleep disturbance.^1^ Over the last decades, depressive symptoms have increased in the general populations.^2^ Critically, depressive symptoms could predict major depressive disorder (MDD) around 15 years later.^3^ Hence, screening subjects with depressive symptoms in the general population is essential for decreasing the rate, burden, and severity of clinical depression.^4^ In addition, a high conversion rate of depressive symptoms to MDD^3^ and the noticeable health-related and economic burden of depressive problems in the general population^5^ makes it imperative to identify the associated behavioral and brain factors of depressive phenotype.

The human life experience highlights a significant mood impairment after night(s) of sleep disturbances, suggesting a robust link between poor sleep and depressive symptoms.^6^ In particular, meta-analyses indicated that sleep disturbance, and particularly insomnia, are critical factors for developing clinical depression.^7^^,^^8^ Treatment of sleep problems reduces depressive symptoms and MDD,^9^ suggesting that targeting sleep quality is necessary for the management of depressive problems. On the other hand, insomnia/hypersomnia are among the diagnostic criteria of MDD, suggesting a bidirectional association between sleep and depression. Nevertheless, many individuals with sleep problems never develop depressive symptoms, and some patients with depressive phenotype report normal sleep patterns, which makes the interrelationships between sleep disturbance and depressive profile very complex. The potential reasons could be inter-individual “biopsychosocial” variability in terms of genetic vulnerability, emotional distress, anxiety, hyperarousal state, emotion regulation abilities, and coping strategies for stressful life events.^6^^,^^10^ The open questions are 1) whether depressive symptoms can be predicted based on sleep quality at the individual subject level, and 2) which underlying behavioral and brain factors contribute to their associations.

Anxiety is the most prominent mental condition that co-occurs with both sleep disturbance and depression.^6^^,^^11^ Moreover, a growing body of neuroimaging evidence highlighted the role of structural and functional brain alterations, mainly in the default mode and salience networks, on the interplay between sleep and depressive symptoms.^12^ Using the Human Connectome Project in young adults (HCP-Young) dataset, Cheng and colleagues^13^ demonstrated that increased functional connectivity between several brain regions mediates the association between depressive symptom severity (DSS) and sleep quality. The volume of the Dentate Gyrus/CA4 Hippocampal subfield could also mediate the association between sleep quality and depressive symptoms in the young healthy subjects.^14^ Importantly, the abnormality of regional GMVs has been introduced as a significant indicative feature of MDD,^15^ and even subclinical depressive symptoms.^16^ Furthermore, a meta-analysis revealed that reduced GMV is an essential characteristic of the first episode of MDD.^17^

Most of the existing behavioral and neuroimaging studies on the link between sleep and depressive symptoms are based on group comparisons and/or correlations using a single sample. Hence, the replicability of observed associations and their generalization to new samples remains an open issue. Thus, the “real world” challenge is the prediction of depressive symptoms in unseen data or independent samples to achieve generalization to future cases that cannot be answered in conventional statistical approaches based on a single sample (i.e., cohort). Advanced machine learning (ML) predictive models increase the hope of identifying the role of neurobehavioral factors in predicting depressive problems across various general population samples, which is crucial for precision medicine and ultimately guiding clinical practice.^18^ Multivariable ML approaches can identify complex (predictive) patterns in brain-behavior associations at the individual level in general populations, which can be replicable and generalizable in other independent cohorts.^19^ Thus, the critical questions of this study are whether and how could sleep quality, anxiety problems, and GMV explain DSS, and how much can ML techniques get this neurobehavioral explanation close to the standard scales of DSS. Without modern statistical tools like ML, we cannot answer these questions and find complicated patterns of depressive symptoms at individual subject level.

Aiming to address the mentioned gaps in the literature, we applied the ML approach in the HCP-Young dataset to predict DSS based on sleep quality, anxiety problems, and the brain's gray matter volume (GMV). In addition, we assessed the role of functional brain measurements i.e., regional homogeneity (ReHo) or fractional amplitude of low-frequency fluctuations (fALFF) in the complementary analyses. Based on the trained ML models in the HCP-Young dataset, out-of-cohort validation of our ML algorithm was conducted on two independent US population-based datasets (i.e., the lifespan Human Connectome Project (HCP-Aging) and enhanced Nathan Kline Institute-Rockland sample (eNKI)) to understand the generalizability of our models across different cohorts. Furthermore, we applied our ML models on a small set of longitudinal subsamples from the eNKI dataset to predict future DSS based on baseline sleep and anxiety data. In addition, we assessed the mediatory role of anxiety and GMV in the association between sleep quality and DSS in the HCP-Young dataset.

## Methods

### Databases

The HCP-Young is a general population dataset acquired by the Washington University-University of Minnesota (WU-Minn HCP) consortium (https://www.humanconnectome.org/).^20^ Their inclusion criteria were to select healthy young adult (22–35 years) participants with no current psychiatric disorder, substance abuse, neurological or cardiovascular disease, or pharmaceutical or behavioral treatment. From all the 1206 participants of the HCP-Young dataset, there were 1113 subjects with sMRI, 1205 subjects had sleep quality scores, 1203 subjects had anxiety scores, and 1198 subjects with DSS scores. In this dataset (our primary sample), we included all participants who had 3 T structural MRI images and phenotypic data that we were interested in this study, i.e., sleep quality, anxiety, and depressive symptoms, and we removed participants who had missing values. Collectively, using this criterion, we included 1101 participants from this dataset. In a complementary analysis, we also removed participants with a lifetime history of diagnosed clinical depression.

The HCP-Aging (https://www.humanconnectome.org/) dataset recruited more than 1200 healthy adults aged 36 to above 100.^21^ However, we could include participants aged 36 to 59 since the DSS questionnaire had been designed for young and middle-aged adults below age 60 (18–59), and there were only DSS scores of participants between 36 and 59 years in this dataset. The eNKI is also a large-scale community-representative dataset of the general population with cross-sectional and longitudinal samples (http://fcon_1000.projects.nitrc.org/indi/enhanced/).^22^ From the eNKI dataset, we included participants (18–59 years) with cross-sectional records for assessment of the generalizability of our ML models and subjects with longitudinal records to see whether the baseline data can forecast future DSS, which is critical to evaluate the long-term effects of sleep quality and anxiety problems on DSS. We included all participants with complete data in the age range of 18–59 from all three datasets.

The ethical approval for each cohort is available on their online documentation. The ethics board of the University Hospital of the Heinrich-Heine University Düsseldorf approved the analysis of these publicly available datasets (No. 4039). However, a study protocol was not prepared, and the study was not pre-registered.

### Behavioral measures

#### Sleep quality

Sleep quality assessment was based on the self-reported Pittsburgh sleep quality index (PSQI) questionnaire,^23^ which has 19 questions assessing sleep quality over the past one month. The PSQI comprises seven components, namely subjective sleep quality, sleep latency, sleep duration, habitual sleep efficiency, sleep disturbances, use of sleep medicine, and daytime dysfunction. The total PSQI score is a sum of these components. Of note, a higher total PSQI score (>5) reflects poor sleep quality.

#### Depressive symptom severity

Depressive symptoms were measured based on the DSM-IV-oriented depressive problems portion of the Achenbach Adult Self-Report (ASR) for ages 18–59.^24^ This questionnaire has 123 items in general, and a total depressive score was obtained from 14 depressive-related items, ranging from 0 to 28 points. A higher score reveals more severe depressive symptoms, and the sex-/age-adjusted t-score above 69 shows clinical depression. Notably, two sleep-related items of this questionnaire were removed in our main ML and mediation analyses. These questions were “I sleep more than most other people during the day and/or night” and “I have trouble sleeping”. We calculated the total score of depressive problems after removing sleep-related items and used this total score in our analyses. Further, as a complementary analysis, we examined the original DSS (we refer to it as DSS'), which involves these two sleep-related items.

#### Anxiety problems

Anxiety score was measured using six relevant items of DSM-IV-oriented ASR for the age range 18–59. None of these items are related to sleep or depressive problems. Similar to DSS, the total score of anxiety has been used in our study, and a higher anxiety score shows more anxiety problems and the sex-/age-adjusted t-score above 69 is the clinical range for anxiety problems.

### Neuroimaging measures

In this study, we used parcel-wise whole-brain GMV to assess the role of brain structure in the link between sleep quality and DSS across three datasets. Further, we assessed resting-state fMRI features (i.e., ReHo and fALFF of the same parcels) in the HCP-Young dataset as a confirmatory analysis (see more details in the supplementary material).

#### Calculation of gray matter parcel volume

T1 structural MRI images were acquired by Siemens 3 T Skyra scanner and preprocessed using the WU-Minn HCP consortium pipelines.^25^ We performed voxel-based morphometry (VBM) using the Computational Anatomy Toolbox (CAT12),^26^ implemented in the Statistical Parametric Mapping (SPM12, https://www.fil.ion.ucl.ac.uk/spm/software/spm12/). During this process, we corrected bias-field distortions, and after noise removal and skull striping, the images were normalized to standard space MNI-152. Then, we segmented the brain tissue into gray matter, white matter, and cerebrospinal fluid. Subsequently, we modulated the gray matter segments for the non-linear transformations performed during normalization to obtain the actual volumes. GMVs of the cortical, subcortical, and cerebellar areas were assessed using functionally-informed in-vivo atlases (400 cortical parcels from Schaefer atlas,^27^ 36 subcortical parcels from Brainnetome,^28^ and 37 cerebellar parcels from Buckner^29^), resulting in 473 brain parcels, as applied previously.^30^

### Statistical analyses

#### Prediction analysis in the HCP-Young dataset

**Fig. 1:**
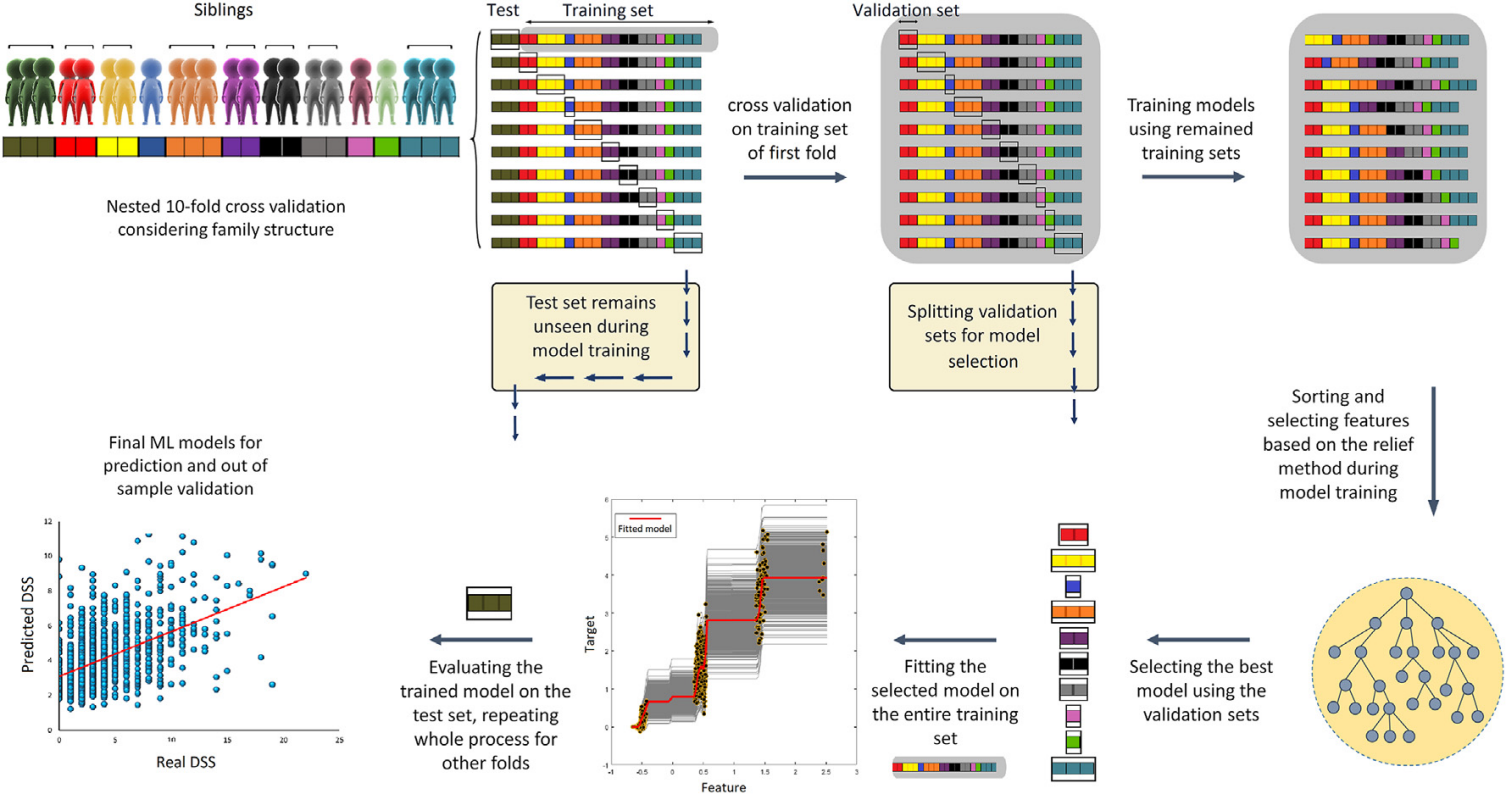
**ML pipeline for prediction of DSS considering family structure in the primary dataset (HCP-Young).** First of all, 10-fold cross-validation was performed so that siblings were not separated in training/test sets. After putting aside the test set (of the first fold from now), we performed a 10-fold cross-validation on the training set (of the first fold) considering family structure. In this stage, we split validation sets and trained models on the remaining training sets. On each fold, we trained models and optimized hyper-parameters ten times with ten different feature numbers. Hence, we had ten folds and ten models for each fold and the algorithm had to select the model with the best performance and minimum error across all folds. Subsequently, the selected model was fitted on the entire training set and then evaluated on the test set. This process repeated for all other nine folds (Note: all units in the figure are arbitrary, DSS: depressive symptoms severity after excluding two sleep-related items).

Ensemble decision tree methods were employed to structure predictive models using MATLAB R2020a software. Ensemble methods of these models were LS-boost and bagging, which were applied as a hyperparameter to be selected automatically by the algorithm (see below). First, we performed nested 10-fold cross-validation considering the family structure of subjects, in which twins and siblings were not separated in the training, validation, and test sets to avoid potential data leakages. We used training sets to construct models, validation sets to select hyperparameters and feature numbers, and unseen test sets to finally evaluate the models’ performance (Fig. 1). Subsequently, regression models were made to regress out age, sex, and total GMV from features of training sets, and then, these models were used for regressing out these covariates from test sets. Then, features of training sets were ranked and sorted (from the maximum importance to the minimum importance) by the relief method to enable the algorithm to select features based on the maximum rank.^31^ After putting aside the validation sets, models were constructed and trained in each remaining training set ten times by ten different feature numbers so that the number of features could also be selected automatically based on the minimum error of prediction of the validation sets. In this stage, hyperparameters were optimized using the Bayesian method,^32^ with 100 iterations. Then, models with the minimum error of prediction of validation sets were selected and fitted on the entire training sets (training + validation) and finally used to predict unseen test sets. Thus, in the end, we had ten models (one model for each test set), and our ML pipeline could select different algorithms LS-Boost/bagging along with its hyperparameters and different feature numbers for each fold. These predictive models had 19 input features consisting of PSQI questions. Subsequently, we added anxiety (total score) and 473 whole-brain GMV features to measure the role of anxiety and GMV in DSS prediction. Of note, against models with a combination of features of GMV, we did not perform a feature section for models with just sleep quality and/or anxiety features because the number of features was not too high, and therefore, feature selection was not necessary. More details of these ML analyses, hyperparameters, and feature numbers are provided in the supplementary materials and the codes are available in the following link (https://github.com/Mahnaz-Olfati/Depression-Prediction).

### Complementary analyses in the HCP-Young dataset

In several follow-up analyses, we controlled for potential issues to examine the robustness of our findings and to cover different aspects of the interplay between behavioral and brain variables as follows: 1) we assessed the predictability of two other ML models (random forest and simple linear regression) in prediction of DSS (eFig. 1); 2) we assessed correlation between sleep quality features to test feature redundancy (eFig. 2); 3) to test the predictive power of functional brain features in our main predictive ML analyses, we calculated ReHO and fALFF of 473 parcels from resting-state fMRI images (eFig. 3); 4) we assessed the predictive power of anxiety (alone) and the combination of GMV and anxiety features separately (eFig. 4); 5) in order to test multicollinearity between variables, we performed cross-prediction of anxiety and DSS and also tested collinearity between all phenotypic parameters using variance inflation factor (eFig. 5); 6) we assessed the additive role of ethnicity and income as potential confounding variables in predicting DSS (eFig. 6); 7) we removed 103 participants with a lifetime history of diagnosed depression to assess the potential confounding effect of the history of clinical depression (eFig. 7); 8) we used seven components of the PSQI, instead of 19 individual PSQI items to assess prediction power of sleep quality components (eFig. 8); 9) and reported the feature importance of ML predictions (eFig. 9); 10) critically, in order to assess the reverse direction of prediction, we assessed the predictability of sleep quality based on depressive symptoms (eFig. 10), 11) we examined the predictability of sleep quality based on GMV (alone) (eFig. 11); 12) we also used original DSS questionnaire (DSS’) including two sleep-related items (as mentioned earlier, we removed those items in our main analyses) (eFig. 12); and 13) compared the results with and without sleep-related items of the DSS questionnaire (eFig. 13); and 14) we tested the impact of anxiety and GMV in the link between sleep quality and DSS by mediation analyses (eFig. 14). Details of these complementary analyses are described in the supplementary material.

### Out-of-cohort validation in two independent datasets

We used two independent large-scale datasets to test whether the results of ML models using the HCP-Young dataset are generalizable to other independent datasets with a broader age range of participants (i.e., the eNKI and HCP-Aging). Therefore, we used the regression model of the primary dataset (HCP–Y) to regress out age, sex, and total GMV in these datasets as well. After training ML models on the HCP-Young dataset, we achieved ten models for each prediction, froze them, and used them to predict individual DSS in the other datasets and averaged the results of all ten models for each participant. Of note, we did not tune our models nor perform cross-validation for these independent datasets to keep the original model parameters steady. Put differently, we used these independent datasets solely for out-of-cohort prediction. All the phenotypic data (sleep quality, anxiety, and DSS) were obtained from the same questionnaires across the three datasets. We also used a subsample of the eNKI dataset (those with follow-up data) to predict future DSS based on baseline sleep quality and anxiety problems. We used the sleep quality and anxiety of their first records as features and the DSS of their second records as the target. Then, we calculated the correlation between the predicted DSS and the DSS of the second record. Finally, as the complementary analyses, we separated participants who had either received or not received neurofeedback therapy intervention between their first and second visits and compared their predictive performance to identify the potential impact of a therapeutical intervention on longitudinal predictions (eFig. 15).

### Mediation analysis

The structural equation modeling (SEM) using Amos 24.0 software^33^ was applied to statistically model the underlying relationship between total sleep quality and DSS scores. In this analysis, a latent variable from brain GMV parcels was calculated and used in the models. Mediation analysis investigates how much of the covariance between two variables can be explained by the mediator variable(s). Age, sex, and total GMV were also controlled in the mediation analyses. More details of mediation analysis are provided in the supplement.

### Role of the funding source

The funders had no role in the design and conduct of the study; collection, management, analysis, and interpretation of the data; preparation, review, or approval of the manuscript; and the decision to submit the manuscript for publication.

## Results

### Demographics

The primary dataset of this investigation (HCP-Young) included 1101 participants (22–35 years, mean age = 28.79 ± 3.69, 54.3% female), 103 of whom (9%) had a history of DSM-IV-based depression episodes during their lifetime. The detailed demographic characteristics of participants are provided in Table 1. We had two other different datasets for out-of-cohort validation analysis i.e., the HCP-Aging and eNKI. We found 378 participants (36–59 years, mean age = 47.3 ± 7, 57.9% female) from the HCP-Aging dataset and 334 participants that had cross-sectional data (18–59 years, mean age = 37 ± 13.8, 62% female) from the eNKI dataset. From the eNKI dataset, we found 66 participants (20–56 years, mean age = 42 ± 9.7, 77.3% female) who had longitudinal records, and there was a 1–5 years gap between the two records across those individuals. Among them, 26 subjects (20–45 years, mean age = 34 ± 8.2, 73.1% female) received neurofeedback therapy between their first and second records, and there was an average of 653 days gap between their records, while the other 40 participants (36–56 years, mean age = 47 ± 6.1, 80% female), who had not received neurofeedback therapy, had an average of 847 days gap between their first and second visits.

**Table 1:** The demographic characteristics of 1101 participants from the HCP-Young dataset, 378 from HCP-Aging, and 334 from eNKI.

Characteristic	HCP-Young No (%)	HCP-Aging No (%)	eNKI No (%)
Age, mean (SD), year	28.79 (3.69)	47.29 (6.96)	37.01 (13.80)
Female	598 (54.3)	219 (57.94)	210 (62.87)
Total GMV, mean (SD) mm^3^	677,142 (66,932)	612,526 (55,469)	602,128 (68,762)
Twin status			
Monozygotic	285 (25.89)	–	–
Dizygotic	170 (15.44)	–	–
Not twin	646 (58.67)	–	–
Pittsburg sleep quality index, mean (SD)			
Total score	4.79 (2.76)	4.62 (2.85)	5.04 (3.06)
Subjective sleep quality	0.89 (0.64)	0.83 (0.69)	1.03 (0.75)
Sleep latency	0.97 (0.82)	0.81 (0.85)	0.95 (0.90)
Habitual sleep efficiency	0.57 (0.82)	0.33 (0.59)	0.63 (0.92)
Sleep duration	0.45 (0.79)	1.21 (1.44)	0.64 (0.88)
Sleep disturbance	1.09 (0.48)	1.01 (0.50)	1.11 (0.52)
Use of sleep medications	0.23 (0.67)	0.36 (0.84)	0.24 (0.67)
Daytime dysfunction	0.59 (0.64)	0.56 (0.63)	0.57 (0.70)
Adult self-report DSM-IV depressive problem scale, mean (SD)			
Raw score	4.14 (3.44)	3.37 (3.42)	2.85 (2.91)
Sex-adjusted, age-adjusted t-score	53.89 (5.69)	–	–
Adult self-report DSM-IV anxiety problem scale, mean (SD)			
Raw score	3.87 (2.67)	3.25 (2.31)	3.64 (2.59)
Major depressive episode			
No	966 (87.74)	–	–
Yes	103 (9.36)	–	–

### Sleep and anxiety predicted DSS in the HCP-Young dataset

**Fig. 2:**
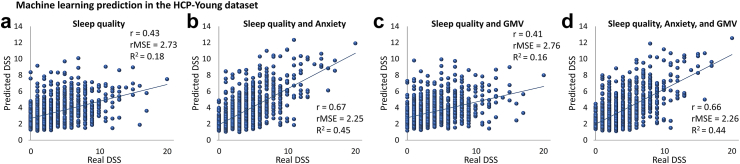
**Prediction of DSS in the HCP-Young dataset.** a) prediction based on sleep quality; b) prediction based on a combination of sleep quality and anxiety problems; c) prediction based on a combination of sleep quality and GMV d) prediction based on a combination of sleep quality, anxiety problems, and GMV (GMV: gray matter volume, DSS: depressive symptoms severity after excluding two sleep-related items, r: correlation coefficient between real and predicted DSS, rMSE: root mean squared error, R^2^: determination coefficient).

The details of ML pipeline for training and evaluation of models in the HCP-Young dataset are presented in Fig. 1. ML models based on sleep quality could predict DSS (r = 0.43, rMSE = 2.73, R^2^ = 0.18) (Fig. 2a). Adding anxiety score to sleep quality features improved the prediction drastically (r = 0.67, R^2^ = 0.45, rMSE = 2.25) (Fig. 2b). Whereas adding GMV features to the sleep quality (r = 0.41, R^2^ = 0.16, rMSE = 2.76) and combination of sleep quality and anxiety (r = 0.66, R^2^ = 0.44, rMSE = 2.26) did not improve their prediction (Fig. 2c and d). For the models with only sleep quality features (i.e., Fig. 2a), we used all 19 scores, since they had no feature redundancy (eFig. 2). Although, the most correlated features of sleep quality scores were the negative correlation between the time of actual sleep and the total sleep quality score (r = −0.58) and the positive correlation between self-estimated sleep quality and the total sleep quality score (r = 0.68) (eFig. 2). However, based on the designed method, the ML algorithm automatically selected different feature numbers (for models with GMV features i.e., Fig. 2c & d) in each fold, the selected ensemble learning method as a hyperparameter for all folds of all models was LS-boost. In addition, the results of random forest and simple linear regression models (eFig. 1) were similar to Fig. 2b. However, we gained a more robust result from the ensemble regression tree.

Our complementary analyses demonstrated that the applied brain morphological and functional features could not significantly predict DSS in general populations (eFig. 3). Removing participants with a history of depression also showed robust predictive results e.g., a combination of sleep quality and anxiety predicted DSS (r = 0.61, R^2^ = 0.37, rMSE = 2.18) (eFig. 7). Moreover, repeating the analyses based on seven components of PSQI (instead of 19 questions of the PSQI) also revealed robust results in predicting DSS (r = 0.64, R^2^ = 0.41, rMSE = 2.32, based on a combination of sleep quality and anxiety) (eFig. 8). The feature importance in the ML model demonstrated that sleep-related daytime dysfunction, sleep disturbance, and subjective sleep quality were more important than other sleep components in predicting DSS (eFig. 9). Importantly, the reverse direction of prediction (prediction of sleep quality based on DSS) revealed a weaker result (r = 0.33, R^2^ = 0.11, rMSE = 2.61) (eFig. 10), indicating the sleep quality might be a better predictor of DSS than the other way around. Further, using the original DSS’ scores (not excluding two sleep-related questions from the depressive questionnaire) provides better prediction results, as expected (e.g., based on a combination of sleep quality and anxiety r = 0.71, R^2^ = 0.50, rMSE = 2.42) (eFig. 12). The mediation analyses demonstrated that those two sleep-related items could explain about 62% of the covariance between sleep quality and DSS’ (eFig. 13). Moreover, we observed that 52.6% of the covariance between sleep quality and DSS can be explained by anxiety, while GMV could not significantly mediate their association (eFig. 14). For more details, see the supplementary file.

### Sleep and anxiety predicted DSS in the independent datasets

**Fig. 3:**
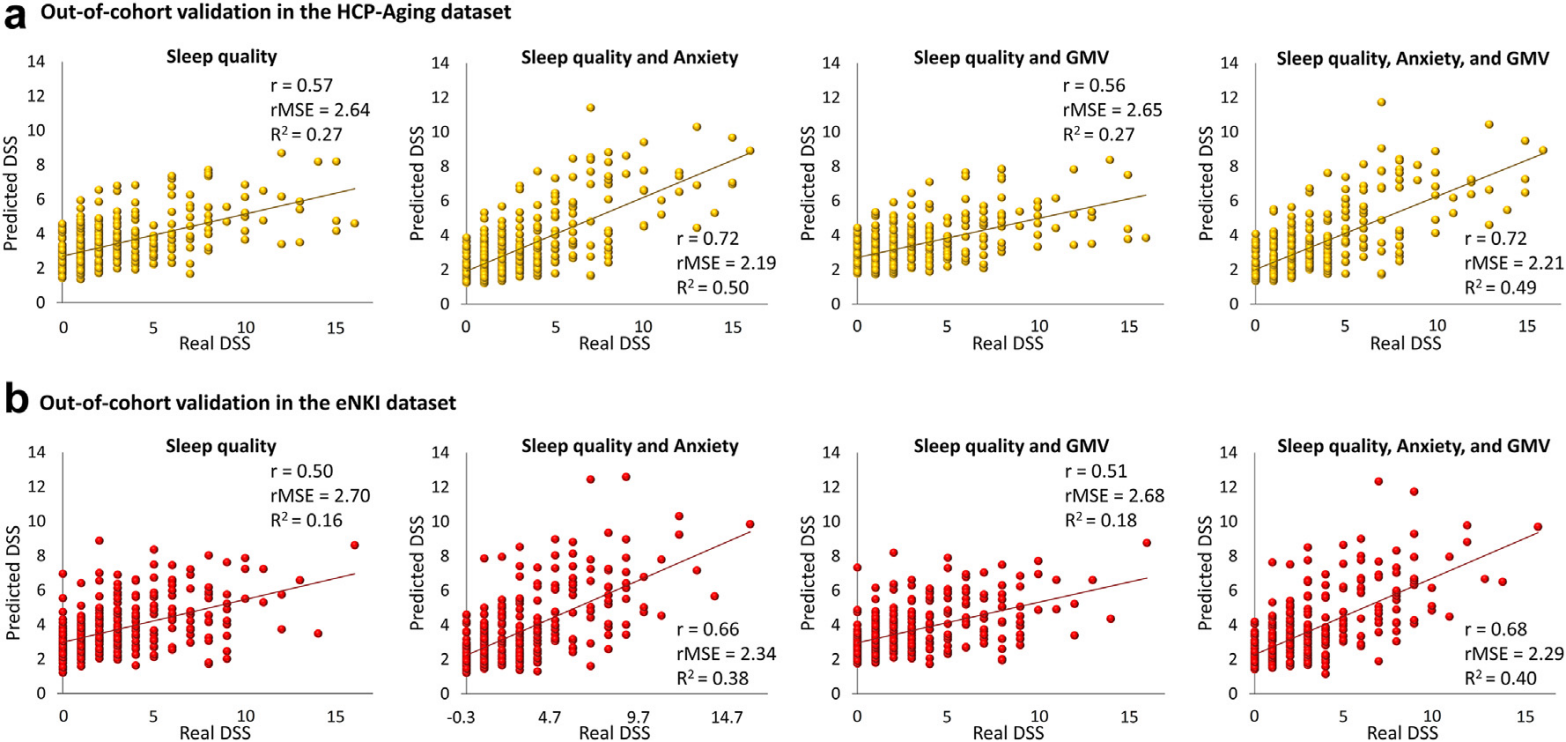
**Out-of-cohort validation of ML results in two independent datasets.** a) prediction of DSS in HCP-Aging dataset based on sleep quality, a combination of sleep quality and anxiety problems, a combination of sleep quality and GMV, a combination of sleep quality and anxiety, and GMV; b) prediction of DSS in eNKI dataset based on sleep quality, a combination of sleep quality and anxiety, a combination of sleep quality and GMV, a combination of sleep quality and anxiety, and GMV (GMV: gray matter volume, DSS: depressive symptoms severity after excluding two sleep-related items, r: correlation coefficient between real and predicted DSS, rMSE: root mean squared error, R^2^: determination coefficient).

Interestingly, we could predict DSS in both HCP-Aging and eNKI cohorts using models that were trained by the HCP-Young dataset (Fig. 3a & b). In the HCP-Aging dataset, sleep quality features could predict DSS robustly (r = 0.57, R^2^ = 0.27, rMSE = 2.64). Further, adding anxiety score to sleep quality features improved the prediction in this dataset (r = 0.72, R^2^ = 0.50, rMSE = 2.19). Adding GMV features to the sleep quality (r = 0.56, R^2^ = 0.27, rMSE = 2.65) and a combination of sleep quality and anxiety score (r = 0.72, R^2^ = 0.49, rMSE = 2.21) provided similar results to the primary dataset.

Similarly, in the eNKI dataset, sleep quality predicted DSS (r = 0.50, R^2^ = 0.16, rMSE = 2.70), and a combination of sleep quality and anxiety scores also predicted DSS (r = 0.66, R^2^ = 0.38, rMSE = 2.34). Adding GMV features to the sleep quality (r = 0.51, R^2^ = 0.18, rMSE = 2.68), and a combination of sleep quality, anxiety, and GMV (r = 0.68, R^2^ = 0.40, rMSE = 2.29) revealed the same result as the HCP-Young dataset.

**Fig. 4:**
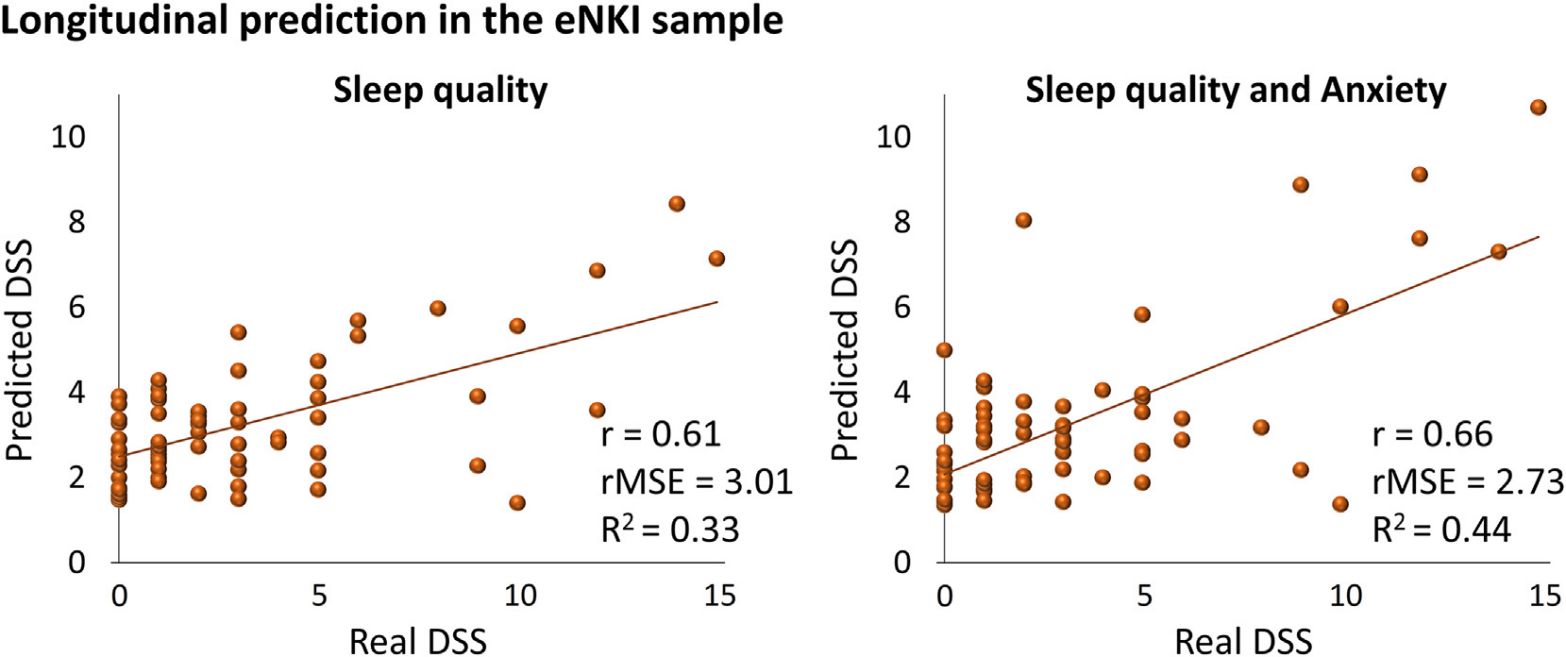
**Prediction of future DSS based onbaselinesleep quality and anxiety.** There were 66 participants in the longitudinal sub-sample of the eNKI dataset (DSS: depressive symptoms severity after excluding two sleep-related items, r: correlation coefficient between real and predicted DSS, rMSE: root mean squared error, R^2^: determination coefficient).

Finally, applying ML models on the longitudinal subsample of the eNKI dataset resulted in the prediction of future depressive symptoms (Fig. 4) based on baseline sleep quality (r = 0.61, R^2^ = 0.33, rMSE = 3.01) and combination of sleep quality and anxiety (r = 0.66, R^2^ = 0.44, rMSE = 2.73). The predictability of DSS in subjects who had not received neurofeedback therapy between their first and second visits was strong (eFig. 15A). Interestingly, ML models could not predict future DSS when participants had received neurofeedback therapy between their first and second visits (eFig. 15B), highlighting the role of intervention on the link between sleep and DSS.

## Discussion

Our findings demonstrated that sleep quality could predict DSS in three independent datasets, and adding anxiety to the sleep quality enhanced such prediction (Fig. 2). The structural and functional brain measurements could not significantly predict DSS or mediate the link between sleep quality and DSS in this study. The ML models provided similar results in two other independent datasets using cross-sectional samples (Fig. 3), suggesting the generalizability of our ML models. Additionally, ML models robustly predicted future individual DSS based on baseline sleep quality and anxiety in a longitudinal subsample (Fig. 4). Our complementary analyses considered the impact of a lifetime history of depression, confounding effects of two sleep-related questions on the depressive assessment, the potential multicollinearity between variables, and predictive performance of the reverse direction suggested the robustness of our main findings. In addition, anxiety could mediate the association between sleep quality and DSS (eFig. 14).

Multivariate ML models can extend conventional statistical approaches and overcome the limitations of existing univariate studies, which resulted in small effect sizes and weak replicability.^34^ To evaluate the generalizability of our results, which are less evident in previous neuropsychiatric studies, we tested the out-of-sample validation in the main cohort and out-of-cohort generalization in two independent cohorts. The recommended gold standard in generalizing ML models is to train a model based on one dataset and then project that trained model on independent populations to gain similar results. Of note, replication means training models again in new independent datasets and achieving similar results.^19^ A recent study provided a replicable ML model, but their model failed to be generalizable in a new independent dataset.^19^ However, our ML models showed successful gold-standard generalization in two external datasets across both cross-sectional and longitudinal levels.

Our findings are consistent with a body of literature showing that sleep disturbance and depressive problems are associated with each other^7^^,^^8^^,^^35^ In large-scale population cohorts, it has also been shown that sleep quality is associated with depressive symptoms.^13^^,^^36^ A recent comprehensive study assessing the role of various lifestyle factors on depression demonstrated that healthy sleep duration was not only the most crucial lifestyle factor in reducing the risk of depressive symptoms in the general population but also decreasing the risk of first depressive episode and treatment-resistant depression.^37^ Longitudinal studies showed that people with sleep initiation problems might experience depression over the next 3–6 years of their life.^38^^,^^39^ Interestingly, toddlers’ sleep problems at the age of 18 months predicted depressive symptoms at the age of 8 years old.^40^ Several large-scale longitudinal studies^41^, ^42^, ^43^ demonstrated that short sleep duration and sleep disturbance should be considered risk factors for developing future depressive symptoms. Although these studies have not used ML models to predict individual DSS within the same sample or other samples, they suggest that poor sleep could be a critical predictor for DSS. A ML study^44^ demonstrated that sleep disorder is one of the most important features to predict depression, particularly in individuals with hypertension. They predicted a binary definition (existence/nonexistence) of depression among adults with hypertension, while our study predicted a continuous (0–28) range of severity of depressive symptoms in three databases. Another large-scale ML-based study found that sleep duration is one of the top five predictors of DSS among home-based older adults.^45^ Lyall and colleagues also applied ML in UK Biobank (n = 64,353) and observed that difficulty getting up, insomnia symptoms, snoring, actigraphy-measured daytime inactivity, and lower morning activity predict depression-related outcomes.^46^ We found that the prediction of DSS based on sleep quality was stronger than the reverse direction. Recently, it has been found that although sleep disturbances have a complex bidirectional relationship with various mental disorders, the most robust observed pathway was the effect of poor sleep in the occurrence of psychiatric conditions.^47^ Our findings support this hypothesis, although we cannot claim any causality between sleep and DSS in the general population samples, as many other factors might influence their association and the design of our study precludes the assessment of the causal pathways in the general population. Thus, the longitudinal role of poor sleep (using both subjective and objective sleep assessments) in developing clinical MDD has to be examined in the future.

In the present study, anxiety problems improved the prediction of DSS based on sleep quality features (Fig. 2). As described in eFig. 4, although anxiety alone could predict DSS with r = 0.62 (the correlation coefficient between real DSS and predicted DSS based on anxiety), the correlation coefficient between anxiety itself and DSS scores was already robust (r = 0.63) (eFig. 13), which could suggest that the ML model based on anxiety features (alone) was not superior. However, while the baseline correlation between sleep quality and DSS was r = 0.29 (eFig. 13), ML models could predict DSS based on sleep quality with r = 0.43 (Fig. 2) which can show the better performance of the ML model based on sleep quality. Thus, it seems that sleep quality is the main predictor of DSS, and anxiety strengths it's prediction. Anxiety scores partially mediated (indirect effect = 69% of the total effect) the link between sleep quality and DSS. The strong interplay between sleep disturbances, anxiety, and depression has been well-documented earlier,^11^ and our study supports such findings. For example, short and long sleep duration are predictors of depression and anxiety in a large cohort.^48^ The additive role of anxiety to sleep in DSS prediction is further supported by the notion that sleep loss increases preemptive responding in the amygdala and anterior insula during affective anticipation.^49^

Previous studies have shown that sleep loss is linked to abnormal activity in the medial prefrontal cortex, amygdala, insula, and anterior cingulate cortex, which were associated with higher levels of next-day anxiety.^50^ One study using the HCP-Young sample indicated that functional connectivity between the lateral orbitofrontal cortex, dorsolateral prefrontal cortex, anterior and posterior cingulate cortices, insula, parahippocampal gyrus, hippocampus, amygdala, temporal cortex, and precuneus mediated the effect of sleep quality on DSS.^13^ Structural brain alterations in the postcentral gyrus and superior temporal gyrus mediate the link between sleep disturbance and depressive symptoms in a small group of shift-working nurses.^51^ Other studies observed that the GMV of the right insula mediates the relationship between sleep quality and anxiety/depressive symptoms among young students.^52^ However, these studies have mainly assessed the simple correlation between sleep quality and depressive symptoms and the brain structural and functional features and have not focused on inter-individual prediction. In the present study, GMV did not significantly predict DSS in any dataset, could not improve prediction performance when combined with sleep and anxiety features, and could not significantly mediate the link between sleep and DSS. One explanation could be the link between sleep disturbance and depressive symptoms anchored in the functional level rather than GMV.^12^ However, our complementary analyses showed that local features of the brain function (i.e., ReHo and fALFF) also could not predict DSS. Although a previous study found that functional connectivity across the brain is a better predictor for behavioral measures than structural and diffusion features, they did not assess the predictability of depressive problems.^53^ In our study, the brain measurements were associated with sleep quality (eFig. 13) but could not predict or mediate DSS and were not correlated with DSS, which according to the amount of sleep quality (mean = 4.79, SD = 2.76 the average of total scores is close to the threshold of poor sleep quality “5”) and DSS (mean _t-score_ = 53.89, SD _t-score_ = 5.69 the average of t-scores is far less than the clinical threshold “69”) in the HCP-Young dataset (Table 1), in average the level of DSS scores in this dataset might not be so prominent to be appearing in our brain structural and functional measurements. Another key point of this study is that we excluded two sleep-related items from the DSS questionnaire. As it is shown in eFig. 13, when we included sleep-related items, the association between sleep quality and DSS score increased. We found some brain areas correlated with DSS scores (similar to previous studies), which could mainly be due to those sleep-related items of depressive problems questionnaire. This might explain the reason why some earlier studies that have used depressive measurements, including sleep-related items, have found brain areas correlated with depressive symptoms. While GMV, ReHo, and fALFF are well-established measures of brain structural and functional properties,^54^ in this study, we did not find evidence that these features could predict DSS or sleep quality or improve their association. The brain-related results of our study were similar to those of existing large-scale neuroimaging meta-analysis studies, which did not find a robust regional abnormality in clinical insomnia disorder, MDD, and late-life depression.^55^, ^56^, ^57^ Although our study focus was to assess depressive symptoms in healthy people rather than MDD, our results were similar to the ML classification models, which could not separate healthy individuals from subjects with insomnia based on brain volumes^58^ or to differentiate healthy individuals from patients with depression based on brain structural and functional values,^54^ indicating that the neurobiological underpinning mechanism of sleep disorders and depression is very complex and needs further elaboration. For instance, it has been shown that genomics, epigenetic mechanisms, and neurotransmitters have a determinant role in the development of depression,^59^ and there are also many other factors affecting depression, which calls for more research using modern computational methods in the future.

The findings of this study have to be seen in light of some limitations. The first is the sample size. We had access to a limited number of general population databases with the same neurobehavioral measures in this field, which global attitudes towards open data and international data sharing consortiums such as ENIGMA-Sleep and ENIGMA-MDD can deal with this problem.^60^^,^^61^ The second limitation concerns the data availability in the datasets. There might be a considerable number of confounders that affect depressive symptoms or their link with sleep quality, which are not assessed in our study due to the lack of the data. In addition, in the case of neuroimaging results, there might be methodological limitations from the data acquisition to data analyses, which could largely affect the results of this paper. The brain measures of this study (GMV, ReHo, and fALFF) might be a coarse-grained assessment of brain structural and functional properties. Hence, the challenge of undecidability also remains here, which concerns whether the lack of predictability of DSS based on our brain measures is due to an actual poor relationship or because our brain measures are not comprehensive enough. Further, large-scale longitudinal datasets are needed to evaluate the long-term predictability of DSS and comprehensively assess the impact of interventions in this field.

In conclusion, we found that sleep quality could predict DSS across cross-sectional and longitudinal samples. Anxiety problems, rather than brain features, improved the performance of the predictive model and mediated the link between sleep and DSS. Although the sample size of our longitudinal analyses was small, our ML models have consistently shown the generalizability of their outcomes in different independent databases. Future large-scale cross-sectional and longitudinal datasets are needed to assess the role of sleep and anxiety on the development of depressive symptoms and clinical MDD in the general population. We hope that our findings incentivize clinicians to consider the importance of screening and treating subjects with sleep disturbance and anxiety problems to reduce the burden of depressive symptoms in the general population.

## Contributors

Conception and study design: M.O., S.B.E. and M.T. Preprocessing and data analysis: M.O., F.S., S.F., S.M.B., K.P. Interpretation M.O., S.G., S.B.E., and M.T. Paper writing and editing: all authors.

## Data sharing statement

We used the public datasets from the Human Connectome Project (https://www.humanconnectome.org/) and the enhanced Nathan Kline Institute-Rockland sample (eNKI) (http://fcon_1000.projects.nitrc.org/indi/enhanced/) and the HCP-Aging dataset (https://fcon_1000.projects.nitrc.org/indi/enhanced/access.html).

## Declaration of interests

The authors declare no conflicts of interest.

## Appendix A Supplementary data

eFigures
